# Trends and the Age–Period–Cohort Effects on Mortality of Breast Cancer Among Iranian Women from 1990 to 2021

**DOI:** 10.34172/aim.34723

**Published:** 2025-09-01

**Authors:** Fatemeh Jafari, Soheila Khodakarim, Abbas Rezaianzadeh, Hamed Karami

**Affiliations:** ^1^Student Research Committee, Shiraz University of Medical Sciences, Shiraz, Iran; ^2^Department of Biostatistics, School of Medicine, Shiraz University of Medical Sciences, Shiraz, Iran; ^3^Colorectal Research Center, Shiraz University of Medical Sciences, Shiraz, Iran

**Keywords:** Age-period-cohort effect, Breast cancer, Joinpoint regression analysis, Mortality

## Abstract

**Background:**

The Eastern Mediterranean Region, including Iran, has the highest incidence and mortality of breast cancer (BC) in women. This study examined changes in BC mortality trends among Iranian women by age period cohort (APC) from 1990 to 2021.

**Methods:**

BC deaths and population by age (1990‒2021) were collected from the 2021 Global Burden of Disease (GBD) study and the average annual percentage change (AAPC) and relative risks (RRs) analyzed by joinpoint regression and the APC model.

**Results:**

From 1990 to 2021, the crude and adjusted BC mortality rates showed an increasing (AAPC=0.913%; 95% CI: 0.436%, 1.393%) and decreasing (AAPC=-0.384%, 95% CI: -0.759%, -0.008%) trend, respectively. The APC analysis exhibited an increasing trend in age effect except in the 60‒74 and 80‒84 age group. The period effect also presented an increasing trend from 1992 (RR=0.716; 95% CI: 0.697, 0.734) to 2021 (RR=1.410; 95% CI: 1.381, 1.441). Additionally, the cohort effect illustrated that the mortality rate decreased consistently from the earlier birth cohort to the later birth cohort (Coef=1.017 in<1901 cohort versus Coef=-0.928 in 2002-2006 cohort).

**Conclusion:**

Between 1990 and 2021, BC mortality in Iran showed an increase in crude rates but a decline in age-adjusted rates, with rising age and period effects and a decreasing cohort effect. These patterns may reflect improvements in early detection and treatment alongside demographic shifts, highlighting the need for continued monitoring to guide control strategies.

## Introduction

 Cancer is a major global health problem, killing more than 70 million people worldwide since 2010.^1^ In 2020, approximately 19.3 million people will be newly diagnosed with cancer worldwide (18.1 million excluding non-melanoma skin cancer), and approximately 10 million cancer-related deaths will occur (9.9 million people excluding skin cancer).^2^ Cancer is also reported to be responsible for 684,996 cancer deaths, and the age-standardized incidence rate and age-standardized mortality rate (ASMR) of cancer rank first and second, respectively.^2^ In Asia, breast cancer (BC) is the most common cancer and the second leading cause of cancer-related death among women, with Asia accounting for 39% of all BC cases globally. This increase in BC mortality across Asia are straining oncology systems and impacting people’s quality of life.^3^ In the Eastern Mediterranean Region (EMRO), BC has the highest incidence and mortality rates compared to other cancers in women.^4-7^ According to IARC, in Iran, BC mortality rate in 2022 was 12.5 per 100,000 people.^8^

 Long-term data from key sources allows us to measure time changes in rates of interest over time and helps identify important risk factors. Trend analysis can also propose new hypotheses or validate the existing ones.^9^ Analysis of age-related disease incidence that ignores the effects of cohort and period can lead to incorrect conclusions.^10^ Age-period-cohort models (APCs) provide additional useful insights by documenting changes in the incidence and mortality of cancer that may be due to age, period time, and birth cohort.^11^ In this model, the age effect reflects changes associated with biological aging, the period effect represents factors that affect all age groups during a specific time period, and the cohort effect illustrates variations between individuals born in the same year or generation. Epidemiological studies using the APC model have improved our understanding of the stress and pathogenesis of several types of cancer.^12^ In Iran, where rapid demographic transitions and changes in cancer risk factors have occurred in recent decades, applying the APC model can help disentangle these effects. However, it is not clear how much this model has been used to study BC mortality trends in the country, which shows the potential value of the present study. This ecologic study was conducted to identify changes in BC mortality based on APC among Iranian women from 1990 to 2021, with the aim of providing evidence on BC prevention and control.

## Materials and Methods

###  Study Data 

 The Global Burden of Disease (GBD) 2021 study, coordinated by the Institute for Health Metrics and Evaluation (IHME), provides comprehensive estimates of mortality for 288 causes across 204 countries and territories from 1990 to 2021.^13^ Its data are accessible via the GBD Results Tool on the Global Health Data Exchange (GHDx) platform, a project organized by the Institute for Health Metrics and Evaluation at the University of Washington^14^ and aggregates diverse data sources, including vital registration systems, surveys, censuses, and disease registries. Data for our ecological study included the number of BC deaths and the female population of Iran, recorded in the GBD by five-year age groups for the period 1990–2021.

###  Joinpoint Regression Model

 First, we calculated ASMR using the direct standardization method, with the INDEPTH^15^ standard population as the reference which is related to low- and middle-income countries. The age-specific weights were derived from the age distribution defined in this population. We selected this standard because Iran is classified as a middle-income country, thus selecting this population offers a more appropriate reference for comparison. To identify changes in mortality rate trends, join point regression was estimated using the Join Point Regression Program version 5.0.2. The joinpoint regression model for the observations, (x_1_, y_1_),…, (x_n_, y_n_), where x_1_ ≤ … ≤ x_n_ without loss of generality, may be written as:


$$E\left[y|x\right]=\beta_{0}+\beta_{1}x+\sigma_{1}{\left(x-\tau_{1}\right)}^{+}+\ldots +\sigma_{k}{\left(x-\tau_{k}\right)}^{+}$$

where the τ_k_’s are the unknown joinpoints and a ^+^ = a for a > 0 and 0 otherwise.

 In short, by using mortality rates as an input, this method identifies the year(s) in which trend changes are created, calculates the annual change (APC) in rates between trend change points, and also estimates the average annual percentage change (AAPC) over the entire study period. The APC from year x to year x + 1 is:


$$APC=\frac{e^{b0+b1\left(x+1\right)}-e^{b0+b1x}}{e^{b0+b1x}}*100=\left(e^{b1}-1\right)*100$$

When there are no joinpoints (i.e. no changes in trend), APC is constant, so it equals the AAPC. The joinpoint regression model assumes that the relationship between time and the log-transformed rates is linear across each segment, that residuals are independent and normally distributed with constant variance, and the joinpoints reflect actual changes to trend rather than random variation. The optimal number of joinpoints was selected using the permutation test with Monte Carlo resampling at a 5% significance level with Bonferroni adjustment.^16^

###  Age-Period-Cohort Analysis

 We performed an APC analysis to determine the effects of age, period, and cohort on the temporal trend of BC mortality in the study period. The APC regression model follows a Poisson distribution and is shown with the following equation:


$$\begin{array}{l} Y_{ij}=µ+\alpha_{i}\text{+}\beta_{j}\text{+}\gamma_{k}\text{+}\varepsilon_{ij}\\ i=1,...,a\\ j=1,...,p\\ k=i+j-1. \end{array}$$

where the response variable *Y*_ij_, is mortality in cell (*i,j*) in the *i*-th row and *j*-th column, *μ* is the intercept and ε *ij* is random errors that are defined for each year and age group, *α*_i_, *β*_j_, and *γ*_k_ are the *i*-th age, *j*-th period and *k*-th cohort effects, respectively.^17^ From < 1901 to 2002‒2006, data were stratified into 22 birth cohorts, a 6-year calendar period from 1992‒1996 to 2017‒2021, and a 17-year age group from 15‒19 to > 95 years. The exact linear relationship among age, period, and cohort (Cohort = Period – Age) leads to a complete collinearity problem, known as the identification problem, which makes it impossible to estimate their unique effects using conventional regression models. To overcome this, we applied the Intrinsic Estimator (IE) method proposed by Yang et al,^18^ which uses a principal component approach to provide unbiased and statistically efficient estimates of the three effects simultaneously. The command for this analysis is “apc-ie” in the STATA software. The coefficients were calculated in the exponential value (exp (*coef.*) = *e*^coef.^) indicating the relative risk (RR) of incidence and mortality of a particular age, period or birth cohort relative to any average level.^19^ The Poisson APC model assumes that the variance of the outcome is equal to its mean. In interpreting the findings, the potential presence of overdispersion and the adequacy of model fit were taken into consideration by information criteria such as the Akaike information criterion (AIC) and the Bayesian information criterion (BIC). Statistical significance was determined at the 5% level (*P* < 0.05) for model coefficients and overall effects.

## Results

**Figure 1:**
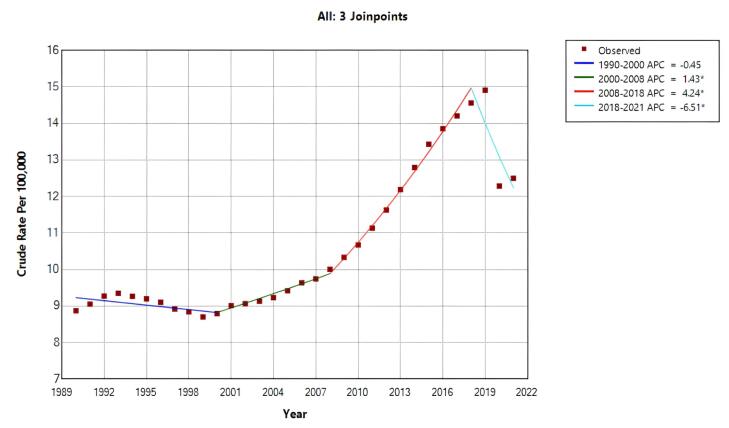
Joinpoint Analysis of Crude (a) and Age-Standardized (b) Mortality Rates of Breast Cancer Among Iranian Women During 1990-2021

 The overall crude mortality trend increased during the entire period (AAPC = 0.913%; 95% CI: 0.436%, 1.393%; *P* < 0.001). However, the trend was not uniform across time. joinpoint regression identified four distinct segments: there was a nonsignificant decrease (APC = -0.451%; 95% CI: -0.999%, 0.100%; *P*= 0.103) during 1990 to 2000; between 2000 to 2008, it increased by 1.433% (95% CI: 0.476%, 2.399%; *P* = 0.005) annually; during 2008 to 2018, it increased by 4.240% (95% CI: 3.567%, 4.917%; *P* < 0.001); and from 2018 to 2021, it decreased by -6.513% (95% CI: -9.771%, -3.138%, *P* < 0.001) annually (Table 1, Figure 1a).

**Table 1:** Crude and Age-Standardized Mortality Trend of Breast Cancer Among Iranian Women During 1990‒2021 Based on Joinpoint Regression

**Variable **	**Years**	**APC (95%CI)**	***P *** **value**	**AAPC (95%CI)**	***P *** **value**
Crude rate	1990‒2000	-0.451 (-0.999, 0.100)	0.103	—	—
	2000‒2008	1.433 (0.476, 2.399)	0.005	—	—
	2008‒2018	4.240 (3.567, 4.917)	< 0.001	—	—
	2018‒2021	-6.513 (-9.771, -3.138)	< 0.001	—	—
	1990‒2021	—	—	0.913 (0.436, 1.393)	< 0.001
Age standardized mortality rate	1990‒2007	-0.105 (-0.340, 0.130)	0.366	—	—
	2007‒2018	1.531 (1.000, 2.066)	< 0.001	—	—
	2018‒2021	-8.571 (-11.600, -5.438)	< 0.001	—	—
	1990‒2021	—	—	-0.384 (-0.759, -0.008)	0.045

 The overall ASMR trend was decreasing during the study period (AAPC = -0.384%, 95% CI: -0.759%, -0.008%, *P*= 0.045). However, joinpoint regression revealed two distinct periods with significant changes: between 2007‒2018, ASMR increased annually by 1.531% (95% CI: 1.000%, 2.066%; *P* < 0.001), followed by a sharp annual decrease of –8.571% from 2018 to 2021 (95% CI: –11.600%, –5.438%; *P* < 0.001) (Table 1, Figure 1b).

**Figure 2:**
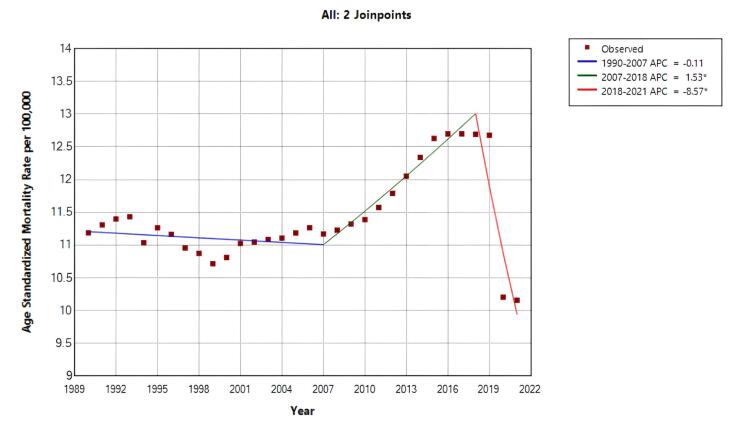
Mortality rate of breast cancer among different age groups of Iranian women from 1990 to 2021

Figure 2 shows the trend of BC mortality changes in different age groups from 1990 to 2021. For almost all age groups, the mortality rate decreased from 1992 to 2001 and increased from 2001 to 2016. From 2016 to 2021, a decreasing trend was observed again, except for the age group ≥ 80 years.

**Figure 3:**
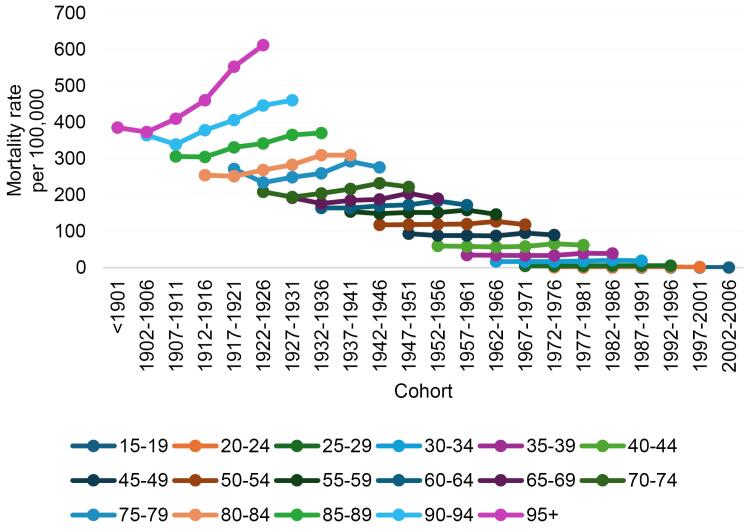
Cohort Trends of Mortality Rate of Breast Cancer in Among Different Age Group of Iranian Women

Figure 3 shows cohort trends in BC mortality in different age groups. Among people aged 80 years or older, younger cohorts had higher mortality rates than older cohorts and in people younger than 79 years of age, younger cohorts had higher mortality rates, but in the final cohort, a decline was observed in all age groups.

**Figure 4:**
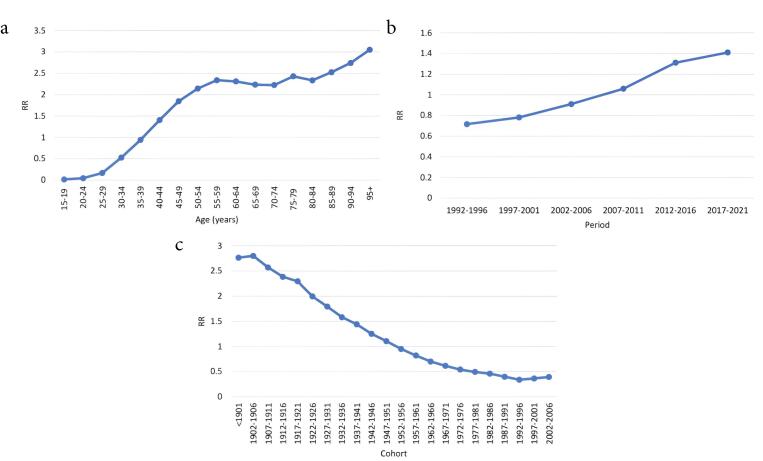
Breast Cancer Mortality of Iranian Women Relative Risks Due to **(a)** Age; **(b)** Period; and **(c)** Cohort Effects

 We used the APC model with the IE method to estimate the coefficients for age, period, and cohort effects. After controlling the period and cohort effects, the age effect exhibited an increasing trend, except in the 60‒74 and 80‒84 age group (Figure 4a). For example, as presented in Table 2, the estimated age effect coefficient for the 15–19-year age group was −4.165 (95% CI: -4.381, -3.949), whereas it was 1.115 (95% CI: 0.970, 1.260) for the ≥ 95-year age group. The corresponding relative risk, obtained as *e*^(1.115-(-4.165))^, indicated that, after adjusting for period and cohort effects, the BC mortality rate in the ≥ 95-year group was approximately 196.36 times that of the 15–19-year group and this interpretation can be used to compare each both age groups. Additionally, the period effect presented an increasing trend from 1992 (RR = 0.716; 95% CI: 0.697, 0.734) to 2021 (RR = 1.410; 95% CI: 1.381, 1.441) (Table 2, Figure 4b). Moreover, the cohort effect illustrated that the mortality rate decreased consistently from the earlier birth cohort to the later birth cohort after adjusting the age and period effects (Coef = 1.017 in < 1901 cohort to Coef = -0.928 in 2002‒2006 cohort), but from the 1997‒2001 group onwards, the situation was reversed (Table 2, Figure 4c). All these analyses were significant at the 0.05 level.

**Table 2:** APC Model Analysis Results of Breast Cancer Mortality Among Iranian Women

**Factor**	Coef. (log RR)^*^	**SE**	**95% CI**	***P *** **value**
Age (years)				
15‒19	-4.165	0.110	-4.381, -3.949	< 0.001
20‒24	-3.121	0.062	-3.244, -2.998	< 0.001
25‒29	-1.799	0.038	-1.874, -1.723	< 0.001
30‒34	-0.641	0.028	-0.697, -0.585	< 0.001
35‒39	-0.062	0.024	-0.110, -0.015	0.010
40‒44	0.339	0.021	0.297, 0.381	< 0.001
45‒49	0.612	0.018	0.575, 0.649	< 0.001
50‒54	0.762	0.017	0.729, 0.796	< 0.001
55‒59	0.849	0.016	0.817, 0.880	< 0.001
60‒64	0.837	0.016	0.805, 0.868	< 0.001
65‒69	0.803	0.017	0.769, 0.836	< 0.001
70‒74	0.799	0.018	0.761, 0.836	< 0.001
75‒79	0.887	0.021	0.846, 0.929	< 0.001
80‒84	0.848	0.025	0.798, 0.897	< 0.001
85‒89	0.926	0.030	0.866, 0.986	< 0.001
90‒94	1.008	0.041	0.927, 1.090	< 0.001
+ 95	1.115	0.074	0.970, 1.260	< 0.001
Period				
1992‒1996	-0.334	0.013	-0.360, -0.309	< 0.001
1997‒2001	-0.246	0.010	-0.267, -0.225	< 0.001
2002‒2006	-0.094	0.008	-0.111, -0.077	< 0.001
2007‒2011	0.058	0.007	0.043, 0.074	< 0.001
2012‒2016	0.271	0.008	0.254, 0.289	< 0.001
2017‒2021	0.344	0.011	0.323, 0.366	< 0.001
Cohort				
< 1901	1.017	0.245	0.535, 1.498	< 0.001
1902‒1906	1.029	0.123	0.788, 1.271	< 0.001
1907‒1911	0.944	0.089	0.768, 1.119	< 0.001
1912‒1916	0.869	0.062	0.747, 0.992	< 0.001
1917‒1921	0.831	0.045	0.741, 0.920	< 0.001
1922‒1926	0.691	0.038	0.615, 0.766	< 0.001
1927‒1931	0.584	0.034	0.516, 0.653	< 0.001
1932‒1936	0.459	0.032	0.396, 0.522	< 0.001
1937‒1941	0.364	0.029	0.305, 0.422	< 0.001
1942‒1946	0.224	0.028	0.168, 0.279	< 0.001
1947‒1951	0.101	0.026	0.048, 0.153	< 0.001
1952‒1956	-0.050	0.025	-0.101, -0.000	0.048
1957‒1961	-0.197	0.025	-0.247, -0.148	< 0.001
1962‒1966	-0.353	0.025	-0.403, -0.303	< 0.001
1967‒1971	-0.486	0.026	-0.538, -0.434	< 0.001
1972‒1976	-0.611	0.028	-0.666, -0.555	< 0.001
1977‒1981	-0.705	0.030	-0.765, -0.646	< 0.001
1982‒1986	-0.776	0.033	-0.841, -0.710	< 0.001
1987‒1991	-0.916	0.040	-0.995, -0.836	< 0.001
1992‒1996	-1.079	0.066	-1.209, -0.949	< 0.001
1997‒2001	-1.010	0.126	-1.257, -0.763	< 0.001
2002‒2006	-0.928	0.267	-1.453, -0.404	0.001

* Coef. represents log-relative risks estimated from the Poisson APC model using the Intrinsic Estimator (IE) method; RR can be obtained by exponentiating the coefficients.

## Discussion

 BC is one of the leading malignancies in women, causing significant complications and placing a heavy burden on the health care systems worldwide.^20^ In our study, the trends of AAPC crude mortality rate increased but ASMR decreased during 1990 to 2021. One study found that mortality rates trended downward in most of the 35 countries studied.^21^ A global study found that BC mortality rates increased in only 4 out of 39 countries, and 25 countries were declining, 20 of which were European countries.^22^ Another study showed that although the incidence of BC is low in the EMRO and African regions (AFRO), the number of deaths is higher.^23^ One possible explanation is the low socioeconomic status. Indeed, patients in deprived areas are more likely to be diagnosed with advanced-stage disease, likely due to later presentation and lower mammography screening rates. Women in these areas are less likely to receive surgery, and if they do, they are more likely to undergo mastectomy compared to those in affluent areas.^24^ It is reported that more than half of women with BC in the Middle East are diagnosed with lymph node metastasis at the third and fourth stages of the disease.^25^ Considering that 69% of patients with advanced stage disease die within 5 years of treatment,^24^ one can say that the mortality rate is high in the EMRO and AFRO regions. This explanation also applies to Iran as a member of EMRO.^5,6^ Two studies in Iran have shown that the mortality rate from BC has increased.^26,27^ It is estimated that only 18% of patients with BC are diagnosed at stage I in Iran^28^ and more than 80% are diagnosed at stage II.^29^

 Our study demonstrated that mortality trends in different age groups were increasing, while in people younger than 79 years of age, a decline was observed during 2016‒2021. The increase in deaths among people aged 80 and over between 2016 and 2021 may be due to the COVID-19 pandemic in the final years of this period, and these people were deprived from screening, diagnostic and treatment services due to their age conditions.^30^ A study in Turkey found an increase in the incidence of higher-stage BC from pre-pandemic to pandemic and post-pandemic eras.^31^ A study in Iran showed that BC screening services in Iran experienced a significant decline after the start of the pandemic.^32^ A qualitative study demonstrated the challenges of Iranian women with BC during the COVID-19 pandemic, including reduced access to diagnostic and treatment services, limited availability of chemotherapy, and heightened patient anxiety.^33^ These findings could be in line with the hypothesis that service disruptions could lead to increased mortality.

 As mentioned above, the mortality rates fell for people under 79 years of age. BC deaths may have been underestimated by the record of people dying from COVID-19. In the APC model, these results also confirmed that cancer mortality increased with age. A study in China found that mortality rates continued to rise, especially among those aged 65 and above.^34^ Age has always been considered an important factor in APC analysis, as it may represent consistent extrinsic factors such as increased mutations or cumulative exposure to carcinogens over time that increase the risk of developing cancer.^35^ In addition, BC diagnosed in older patients typically has a poorer prognosis due to increased underlying disease or decreased physical function, and is less responsive to various treatments or less compliant with treatment guidelines. This results in under- or overtreatment.^36^ Moreover, the number of high-risk individuals is expected to increase in the future due to longer life expectancy and rapid aging of the population. Therefore, early screening can be carried out in high-risk individuals to prevent BC.

 After adjusting for age and cohort effects in the APC model, the period effect showed an increasing trend. In other words, BC mortality rates increased from 1992 to 2021. Other studies have concluded that there is an increasing trend in the period effect on BC mortality.^37-39^ The increasing period effect on BC mortality may be attributed to several factors that have changed over time and impacted the entire population. These include shifts in lifestyle such as reduced physical activity, increased prevalence of obesity, and changes in reproductive behaviors like delayed childbearing and lower fertility rates, all of which are associated with higher BC risk.^40^ Environmental exposures, such as endocrine-disrupting chemicals, may also play a role by increasing hormonal risk factors across the population.^41^ Additionally, improvements in cancer registration and diagnosis may have contributed to the observed increase in mortality rates. Asian countries are no exception,^42^ with one study reporting the highest increase in BC incidence,^43^ which is one of the causes of increased mortality. Additionally, the COVID-19 pandemic in recent years was another reason for the increase in BC deaths due to the partial interruption and reduction of public and private screening activities and lack of early detection.^44^

 Cohort effects imply changes in some characteristics among people of a certain age but defined by different years of birth or other common experiences. In Figure 3, mortality trends across birth cohorts appear to increase for more recent birth cohorts; however, after adjusting for the age and period effects in the APC-IE model, the relative risks of mortality were significantly lower for the more recent cohorts compared with the earlier cohorts. This observed difference may be because the cohort effect is confounded by the age or period effects in the traditional method. Possible reasons for this decrease include broader access to diagnostic and treatment services, as well as healthier lifestyles and greater awareness of health and disease prevention in later-born cohorts than earlier-born cohorts.^37,45^ In China, the cohort effect also illustrated a decreasing trend in the relative risk of BC mortality between 1990 and 2015,^46^ whereas in Taiwan, the cohort effect initially increased and then decreased.^42^

## Conclusion

 Our study showed that the crude trend of BC mortality in Iran increased but ASMR decreased during 1990‒2021, and the trends of age and period effects increased despite a decrease in the cohort effect. In summary, it can be said that the disease burden of BC among Iranian women remains a major challenge that requires urgent attention. Public health strategies should focus on increasing awareness about BC prevention, promoting early detection through screening programs, and ensuring timely access to effective treatment to reduce mortality.

 The strength of this study is that it provided the first comprehensive national estimate of BC mortality in Iran over a 31-year period. Furthermore, the novelty of this study lies in the application of advanced trend models to study cancer mortality. However, we also faced some limitations. First, the accuracy of the results was based on GBD study estimates calculated from different data sources, so the results need to be verified by a large epidemiological study. Second, the obtained data lacked information regarding staging, screening, and other specific BC information. Third, the study results cannot be generalized to individuals as this leads to the ecological fallacy.
